# Comparative analysis of serum total IgE levels and specific IgE levels in children aged 6 to 9 years with tic disorder and normal children

**DOI:** 10.1186/s12887-023-04233-5

**Published:** 2023-08-14

**Authors:** Yang Liu, Yanqin Li, Xiuqing Ma, Ling Yu, Yuan Liang, Chunsun Li

**Affiliations:** Department of Respiratory and Critical Care Medicine, the First Medical Center of PLA General Hospital, Beijing, 100853 P. R. China

**Keywords:** Tic disorder, Allergy, Children, Total IgE, Specific IgE, House dust mite, Dust mite

## Abstract

**Objective:**

The study was to investigate serum total IgE levels and the distribution of specific IgE types in children aged 6–9 years with tic disorder, in order to provide knowledge for diagnosis and treatment of children with tic disorder.

**Methods:**

Total serum IgE levels were detected by enzyme-linked immunosorbent assay (ELISA). Specific IgE levels in 72 children with tic disorder and normal 31 children were detected by EUROblot, respectively.

**Results:**

The total serum IgE level of children with tic disorder aged 6–9 years was significantly higher than those of children in control group. Specific IgE distribution in tic disorder group was observed increased mainly including inhaled mugwort, dust mite combination 1 (house dust mite/dust mite), mold combination (penicillium point/mycobacteria/Aspergillus fumigatus/streptomyces), cockroaches in Germany respectively, and also food freshwater fish combination 1 (salmon/sea bass/carp), marine fish combination 1 (cod/lobster/scallop), egg white, and crab, while elevated specific IgE of normal children group was mainly food-based (egg white, milk, and soybean). The significant different specific IgE between two groups was dust mite combination 1 (house dust mite/dust mite) (*P* < 0.05).

**Conclusion:**

The total serum IgE level of children with tic disorder aged 6–9 years was significantly increased, which may be related to the disease. Specific IgE in children with tic disorder was mainly inhalation allergens, especially dust mite combination 1 (house dust mite/dust mite), which should be avoided in clinical diagnosis and daily life.

## Introduction

Tic disorder (TD) is a complex, chronic neuropsychiatric disorder predominating in children and adolescents, which is characterized by rapid, involuntary, sudden, repetitive, non-rhythmic, single or multiple muscle motor tics and/or vocal tics. It may be accompanied by one or more comorbidities, such as attention deficit hyperactivity disorder (ADHD), anxiety disorder (AD), depressive disorder (DD), learning difficulties (LD), obsessive compulsive disorder (OCD), sleep disorder (SD), etc. [1]. Its incidence is more common in school-age children and pre-school children, and it is most common in children aged 5–10 years, with more males than females, with a male-to-female ratio of (3–4):1 [2]. The prevalence of TD has been increased in recent years, which endangered children’s health seriously and brought great pain to their families.

The etiology of TD has not yet been fully clarified. Current research results show that its etiology is related to biological factors (genetic factors, immune factors, neurobiochemical factors, etc.), mental and psychological factors (family atmosphere, educational methods), etc. [3]. The etiology and pathogenesis of tic disorder are important to the diagnosis and treatment of the disease. It was reported that the abnormality of immunity may be related to the pathogenesis of TD in children [4]. The concentration of immunoglobulin E (IgE) in serum is extremely low, accounting for about 0.002% of immunoglobulins, mainly in blood vessels, skin, and mucous membranes. It is produced by plasma cells and can bind to mast cells and basophilic polymorphonuclear granulocytes in the blood. When the allergen interacts with the IgE bound to cells, it promotes the degranulation of cells and releases histamine, thereby causing allergic reactions, such as serum sickness, hay fever and other immediate allergic reactions. Increased IgE is common in allergic asthma, parasitic infection, seasonal allergic rhinitis, pulmonary bronchial aspergillosis, drug allergy, IgE myeloma, liver disease, systemic lupus erythematosus, rheumatoid arthritis and other diseases. Decreased IgE is common in some ataxia telangiectasia, agammaglobulinemia, non-IgE myeloma, chronic lymphocytic leukemia, immune insufficiency and other diseases. Many studies concluded that 20–35% of children with tic disorders are due to their own compromised immune system. Most children often had symptoms such as coughing, throat clearing, and sniffling when they were first diagnosed. The symptoms could be relieved after antiallergic treatment. Therefore, the prevalence of TD might be closely related to allergies. In this study we explored the significance of total IgE in children with tic disorder, and analyzed the distribution of specific IgE by detecting the levels of serum total IgE and specific IgE in children with tic disorder, so as to provide basis for clinical diagnosis and treatment.

## Materials and methods

### Specimen collection

The study was approved by the review board and Ethics Committee of Chinese People’s Liberation Army General Hospital (S2023-119-01). Three mL venous blood of 72 TD patients aged 6–9 years was collected, 58 males and 14 females, with an average age of 7.4 years (7.4 ± 1.2), all from the Children’s Medical Center of the First Medical Center of Chinese PLA General Hospital from January 1, 2018 to December 31, 2019 outpatients. The diagnostic criteria for TD in the American Diagnostic and Statistical Manual of Mental Disorders 4th Edition (DSM-IV) was used [5]. The control group was consisted of 31 children who received health care and physical examination at the Children’s Medical Center of the First Medical Center of Chinese PLA General Hospital during the same period, including 20 males and 11 females, with an average age of 7.1 years (7.1 ± 1.1). The blood specimen was centrifuged at 3000 r/min for 10 min after static settlement, and serum was collected for detection.

### Experimental methods

The enzyme-linked immunosorbent assay (ELX800 automatic microplate reader, American BioTek Instruments Inc) was used to determine serum total IgE levels by EU Medical Diagnostics Total IgE Detection Kit, according to the instructions of suppliers. The EU imprinting method (EURROIMMUN EUROBlotMaster, Germany) was used to determine serum specific IgE levels by EU Medical Diagnostics Inhalation and Food Allergen-Specific IgE Antibody Detection Kit (EU Blot). Values ≥ 0.35–0.7 kU/l correspond to RAST class 1, values ≥ 0.7–3.5 kU/l to RAST class 2, values ≥ 3.5–17.5 kU/l to RAST class 3, values ≥ 17.5–50 kU/l to RAST class 4; values ≥ 50–100 kU/l to RAST class 5 and values ≥ 100 kU/l to RAST class 6, respectively [6].

### Statistical analysis

SPSS22 software was used to analyze the data. The quantitative data of two groups were represented by the median interquartile range M (IQR). The comparison between data of two groups was performed by the Mann-Whitney U test, and the rate comparison was performed by *χ*2 test. *P* < 0.05 was considered statistically significant.

## Results

### Total IgE levels in children with tic disorder

**Fig. 1:**
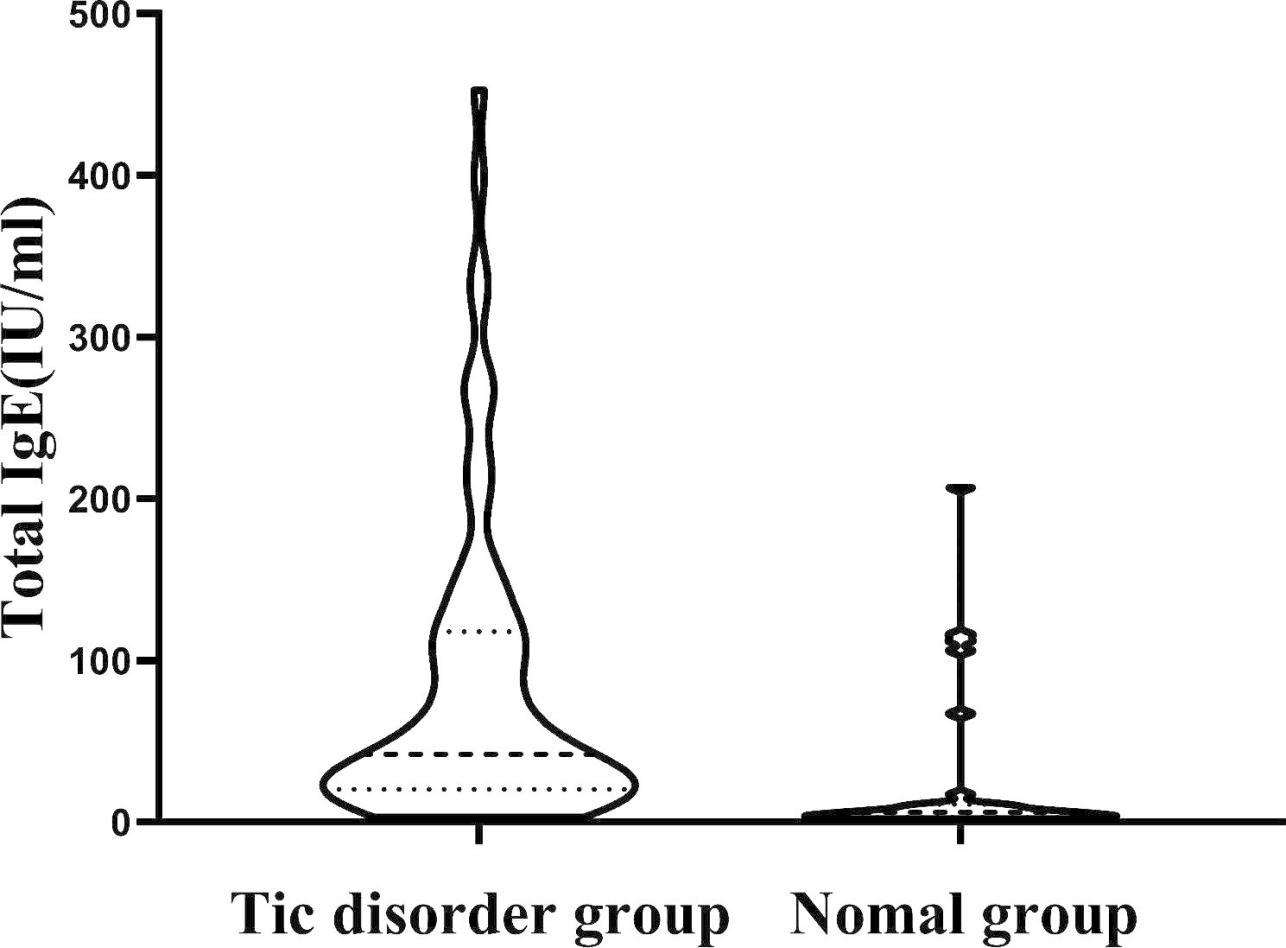
Comparative analysis of serum total IgE levels in children aged 6 to 9 years with tic disorder and normal children. The median and interquartile range of total IgE level of 72 children in TD group were 42 (IQR: 20.25 ~ 117.75), and the median and interquartile range of total IgE level of 31 children in normal group were 6 ( IQR: 3 ~ 11). Mann-Whitney U test was used for the data of two groups, Z=-5.309, *P* < 0.001, the difference was statistically significant

The serum total IgE level of 72 TD children aged 6–9 years was higher than that of 31 control children (Fig. 1). The median and interquartile range of total IgE level of 72 children in TD group were 42 (IQR: 20.25 ~ 117.75), and the median and interquartile range of total IgE level of 31 children in control group were 6 ( IQR: 3 ~ 11). Mann-Whitney U test was used for the data of the two groups, Z=-5.309, *P* < 0.001, the difference was statistically significant. It was suggested that the increase of total IgE level may be related with TD disease.

### Distribution of specific IgE in tic disorder children group

**Fig. 2:**
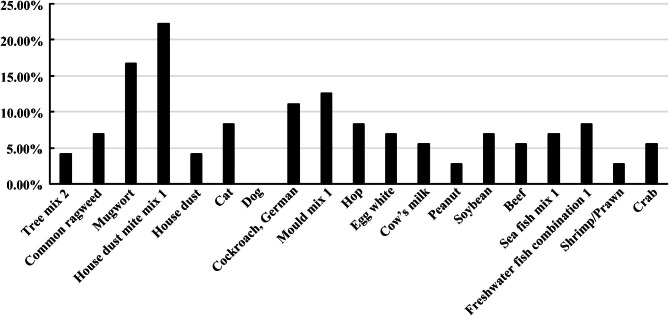
The specific IgE in tic disorder children

The specific IgE increase in 72 cases of 6–9 years old TD children group was observed as inhalation dust mite combination 1 (house dust mite/dust mite) (22.22%), mugwort (16.67%), mold combination (Penicillium punctate/Branches sp./Aspergillus fumigatus/Alternaria) (12.5%), German cockroach (11.11%), hops (8.33%), cat hair (8.33%), common ragweed (6.94%), house dust (4.17%) ), tree combination 2 (willow/poplar/elm) (4.17%), dog epithelium (0%). Food IgE elevation was observed as freshwater fish combination 1 (salmon/bass/carp) (8.33%), egg white (6.94%), soybean (6.94%), marine fish combination 1 (cod/lobster/scallop) (6.94%), milk (5.56%), beef (5.56%), crab (5.56%), peanut (2.78%), shrimp (2.78%). The specific IgE elevation in TD group was mainly inhaled IgE elevation. The results are shown in Fig. 2.

**Fig. 3:**
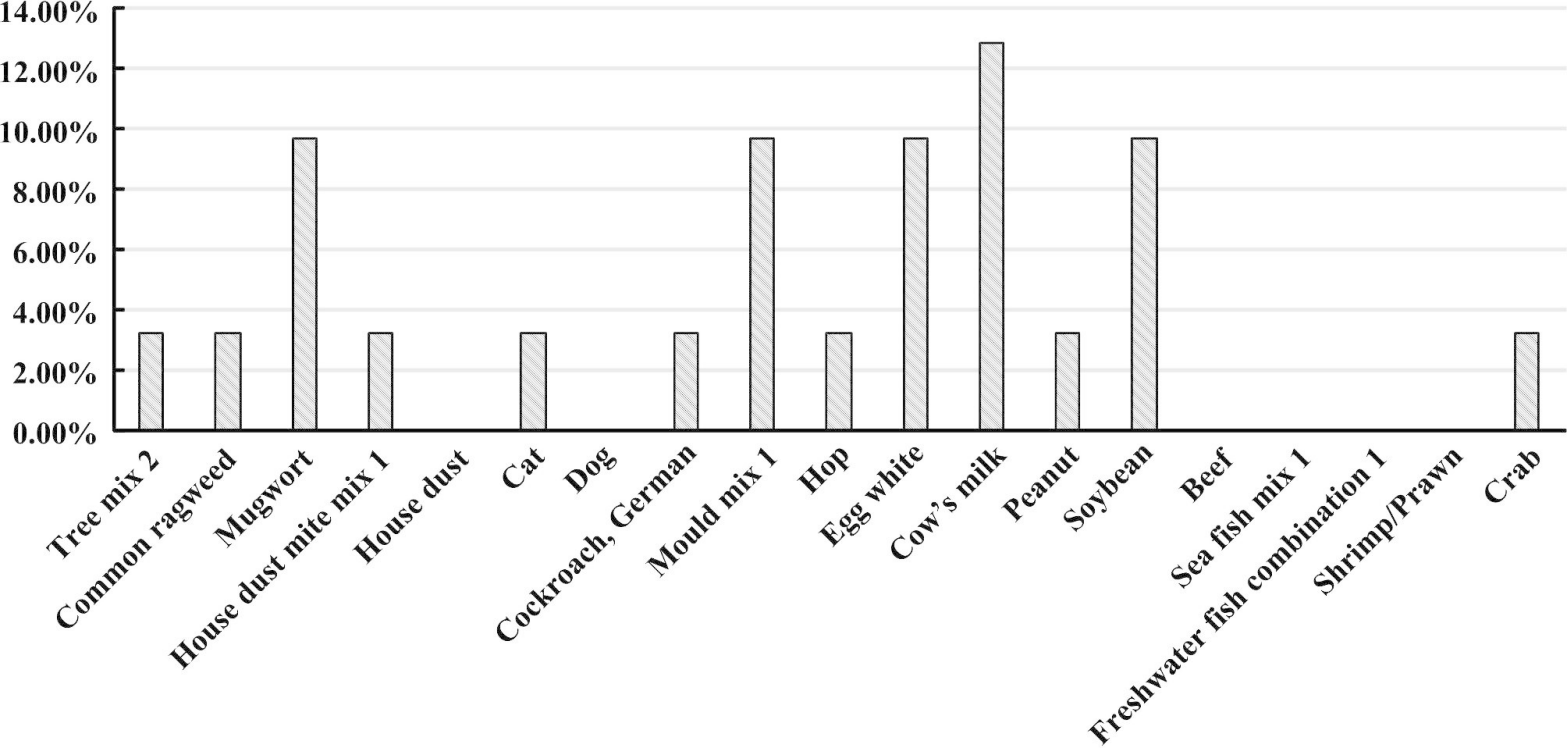
The specific IgE in normal children

The inhalation specific IgE elevation of 31 children in normal control group was showed as Artemisia annua (9.68%), mold combination (Penicillium punctata/branchial sp./Aspergillus fumigatus/Alternaria alternaria) (9.68%), dust mite combination 1 (house Dust Mite/Dust Mite) (3.22%), German cockroach (3.22%), hops (3.22%), cat hair (3.22%), common ragweed (3.22%), tree Combination 2 (Willow/Poplar/Elm) (3.22%), house dust (0%), dog epithelium (0%). Food-induced increases in IgE were showed as milk (12.9%), soybean (9.68%), egg white (9.68%), peanut (3.22%), crab (3.22%), beef (0%), marine fish combination 1 (cod/Lobster/Scallop) (0%), Freshwater Fish Combo 1 (Salmon/Perch/Carp) (0%), Shrimp (0%). The increase of specific IgE in children of control group was mainly food-induced. The results are shown in Fig. 3.

**Fig. 4:**
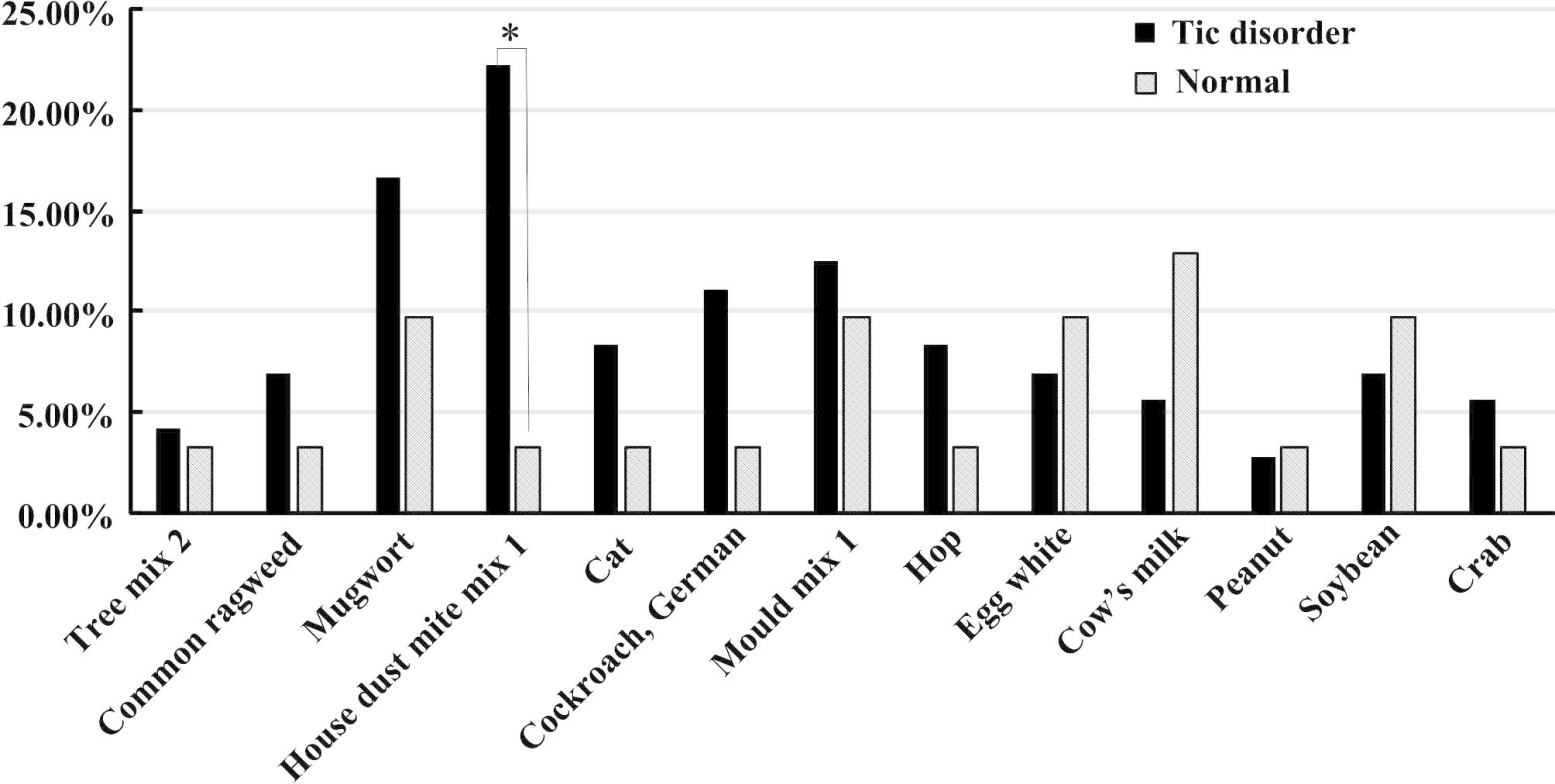
Statistical analysis of the meaning of specific IgE in tic disorder group and normal group. The positive rate of specific IgE levels in tic disorder group and normal group were tested and compared by *χ*2 test. Only dust mite allergen was found statistically significant (*χ*2 = 4.379, *P* = 0.036),*P < 0.05.

The positive rate of specific IgE levels of 72 children aged 6–9 years in TD group and 31 children in control group were tested and compared by *χ*2 test. Only dust mite allergen was found statistically significant (*χ*2 = 4.379, *P* = 0.036). There were no statistical significance in comparison of mugwort, common ragweed, tree combination 2 (willow/poplar/elm), cat hair, German cockroach, mold combination (Penicillium punctata/brachospora/aspergillus fumigatus/alternaria), hops, chicken egg whites, milk, peanuts, soybeans and crabs between two groups. The results are shown in Fig. 4.

## Discussion

In this study, we found that total IgE level of children with tic disorders aged 6–9 was significantly higher than that of normal children (P < 0.05). The specific IgE elevation in TD group was mainly inhaled dust mite combination 1 (house dust mite/dust mite), mugwort, and mold combination (Penicillium punctata/branchial sp./Aspergillus fumigatus/alternariaalternaria). The increase of specific IgE in children of normal group was mainly in milk, egg white and soybean. The comparison of the positive rates of specific IgE between two groups showed that only dust mites had statistical differences, suggesting that dust mites may have a certain significance in the pathogenesis of tic disorders. Whether tic symptoms can be considered as a manifestation of allergic reactions remains to be clarified in further research.

At present, there are few studies on children with tic disorder and serum IgE. In recent years, relevant reports found that the etiology of tics and of allergic diseases have certain similarities [7]. Yuce et al. [8] found that the incidence of tic disorders was significantly higher in patients with allergic diseases, and speculated that allergic diseases and tic disorders shared the same immunological pathogenesis. Chen [9] found that patients with ADHD + tic disorder, ADHD alone, and tic disorder alone were more likely to have allergic diseases than control group. They reckoned that the altered immune reaction and dysregulated cytokine secretion may be the common pathway among those distinct diseases. Zhang’s [10] analysis of the medical and allergy history of tic disorder patients showed that respiratory tract infection, conjunctivitis, surgery/trauma, and history of food and drug allergy were associated with recurrence. Zhang [11] investigated the IgE levels before and after the treatment of Chou-Dong-Ning granules through controlled experiments, and found that Chou-Dong-Ning granules can reduce the level of serum total IgE, which suggested that the occurrence of TD might be related to serum total IgE. Our results found that total IgE level of patients with tic disorder was significantly higher than that of normal control group. The increase in total IgE indicates that allergic reactions may occur in the body. Changes in immune status may cause tic disorder in the body. After further analysis, by comparing the specific IgE distribution, we found that dust mite combination 1 (house dust mite/dust mite) had statistical significance. The increase of dust mite specific IgE may be a potential factor in TD patients. In clinical diagnosis, more attention should be paid to the relationship between dust mites and TD patients. It was speculated that elevated IgE stimulated the body to produce a large number of cytokines, leading to immune pathological damage and causing tic disorders. However, there are currently few reports revealing the relationship between IgE and tic disorder. Our study is a simple validation of the phenomenon discovered in clinical practice and requires further in-depth research. This may be helpful in improving the diagnosis and treatment of tic disorder patients.

At the beginning of 2020, in order to prevent the spread of COVID-19 in China, people began to use masks. The use of masks has changed the impact of inhaled allergens in the environment on children. Using allergen data from January 1, 2018 to December 31, 2019 in this study can more accurately reflect the real situation.

Children with tic disorder frequently occur in the age range of 5 to 10 years. The data collected from children aged 6 to 9 years in this study can also better reflect the real situation of the disease.This study only analyzed the IgE levels of children aged 6 to 9 years, and more research needs to be supplemented in the future due to limited data. In conclusion, this study analyzed the serum total IgE levels and specific IgE levels in children aged 6 to 9 years with tic disorder and normal children, and provided some knowledge to clinical research of tic disorders.

## Data Availability

The datasets used and/or analysed during the current study available from the corresponding author on reasonable request.

## References

[1] Deng Hongzhu,Zou Xiaobing (2012). Children with tic disorder comorbidity. Chin J Practical Pediatr.

[2] Brabson LA, Brown JL, Capriotti MR (2016). Patterned changes in urge ratings with tic suppression in youth with chronic tic disorders. J BehavTher Exp Psychiatry.

[3] Liu F, Ye J, Yao B (2019). Meta-analysis of the relationship between tic disorder and streptococcal infection in children. J Med Res.

[4] Sun X, Wang L (2021). Children’s tic disorder and vitamins a, D and B12 research progress of level. Stud Trace Elem Health.

[5] American Psychiatric Association. Diagnostic and statistical manual of mental disorders. 4th ed (DSM-IV-TR). Washington, DC: American Psychiatric Association; 2000. p. 111–4.

[6] Sander I, Kespohl S, Merget R (2005). A new method to bind allergens for the measurement of specific IgE antibodies. Int Arch Allergy Immunol.

[7] Yildirim Zeynep , Karabekiroglu Koray, Yildiran Alisan (2021). An examination of the relationship between regulatory T cells and symptom flare-ups in children and adolescents diagnosed with chronic tic disorder and Tourette syndrome. Nord J Psychiatry.

[8] Yuce M, Guner SN, Karabekiroglu K (2014). Association of Tourette syndrome and obsessive-compulsive disorder with allergic diseases in children and adolescents: a preliminary study. Eur Rev Med Pharmacol Sci.

[9] Chen M-H, Su T-P, Chen Y-S (2013). Attention deficit hyperactivity disorder, tic disorder, and allergy: is there a link?A nationwide population-based study. J Child Psychol Psychiatry.

[10] Zhang Y, Xiao N, Zhang X (2022). Identifying factors associated with the recurrence of Tic Disorders. Brain Sci.

[11] Zhang F, Feng S (2015). The effect of Chou-Dong- Ning granule on children with multiple tourette syndrome and the effect of serum total IgE. J Changchun Univ Traditional Chin Med.

